# Retinal and choroidal microvascular alterations in Behcet’s disease without ocular manifestations: A systematic review and meta-analysis

**DOI:** 10.3389/fmed.2022.911990

**Published:** 2022-07-22

**Authors:** Shipei Fan, Xingyu Shi, Zhen Chen, Xia Li, Songping Yu, Jun Li

**Affiliations:** ^1^Department of Ophthalmology, Lishui Municipal Central Hospital, The Fifth Affiliated Hospital of Wenzhou Medical University, Lishui, China; ^2^Department of Nephrology, Lishui Municipal Central Hospital, The Fifth Affiliated Hospital of Wenzhou Medical University, Lishui, China

**Keywords:** Behcet’s disease, macula, vessel density, optical coherence tomography angiography (OCTA), meta-analysis

## Abstract

**Purpose:**

We performed a systematic review and meta-analysis to examine the microvascular alterations in non-ocular Behcet’s disease (BD) using optical coherence tomography angiography (OCTA).

**Methods:**

A comprehensive search was performed in Pubmed, Embase and Cochrane databases for eligible studies from inception to February 2022. Detailed clinical demographics were extracted from each study by two independent reviewers. The weighted mean difference (WMD) and 95% confidence intervals (CI) were used to compare the OCTA parameters between non-ocular BD and healthy controls. Stata 12.0 was adopted to conduct statistical analyses.

**Results:**

Ten cross-sectional studies involving 386 eyes in non-ocular BD and 418 eyes in healthy volunteers were ultimately included in the present analysis. When considering superficial capillary plexus (SCP) and deep capillary plexus (DCP), no significant differences of vessel densities in the whole enface image, fovea and perifovea were evaluated between two groups. Significantly reduced parafoveal vessel density of SCP was observed in non-ocular BD in comparison with healthy group (WMD = −1.33, 95%CI: −1.78, −0.89; *I*^2^ = 0.6%), while slightly decreased parafoveal vessel density was assessed in DCP (WMD = −1.47, 95%CI: −3.30, 0.35; *I*^2^ = 89.3%). Significantly increasing foveal avascular zone (FAZ) area was observed in non-ocular BD when compared to healthy controls (WMD = 0.11, 95%CI: 0.03, 0.19; *I*^2^ = 95.3%). There was no significant difference in flow area of choriocapillaris between non-ocular BD and control group (WMD = 0.06, 95%CI: −0.19, 0.32; *I*^2^ = 0%).

**Conclusion:**

Based on current analysis, our results demonstrated significantly lower parafoveal vessel density of SCP and lager FAZ area in full vasculature in non-ocular BD. The retinal microvascular alterations appear before the emergence of ocular manifestations.

**Systematic Trial Registration:**

[https://www.crd.york.ac.uk/PROSPERO/], identifier [CRD42021244856].

## Introduction

Behcet’s disease (BD), first reported in 1937, is a multisystemic autoimmune disorder characterized by recurrent oral and genital ulceration, uveitis, and erythema nodosum (1). Ocular involvement, which occurs in approximately 70% BD patients, is featured by non-granulomatous posterior or pan-uveitis with retinal vasculitis, potentially leading to poor prognosis and irreversible blindness (2). Fluorescein angiography (FA) has been demonstrated to be the gold standard in diagnosing and monitoring the inflammatory activity of BD uveitis. FA is a useful and sensitive tool to show the dye leakage, diffuse vasculitis, optic disc edema, macular ischemia, and retinal neovascularization (3, 4). However, FA may cause side effects and allergic reactions of dye injection, limiting the clinical widespread application.

Optical coherence tomography angiography (OCTA) is a novel non-invasive technique that provides rapid and high-resolution imaging of retinal and choroidal microvasculature separately. OCTA has the capability to differentiate the superficial capillary plexus (SCP), deep capillary plexus (DCP), outer retina, and choriocapillaris, which offers essential insights into the pathogenesis and progression of fundus lesions (5). Moreover, the high repeatability and quantitative analysis of OCTA allows clinical physicians to discover and evaluate the pathological changes and therapeutic effects efficiently. In addition, multiple publications have focused on the microvasculature alterations of BD patients without clinically ocular involvement, yet the conclusion remains controversial.

Previous studies have observed that vessel densities of SCP and DCP decreased in comparison with healthy controls in active and remission BD uveitis, indicating the microvascular disruption of retinal plexus in ocular BD (6, 7). Recent studies also showed significantly decreased vessel densities in non-ocular BD (8, 9), while similar vessel densities between non-ocular BD and control group were identified by Goker et al. (10). A meta-analysis including 6 studies have been demonstrated significantly reduced microvascular perfusion of whole enface, foveal and parafoveal regions in SCP and DCP in non-ocular BD, yet the clinical features of foveal avascular zone (FAZ) and choroidal microvasculature between non-ocular BD and healthy individuals remain unclear (11). To shed light on this issue, this meta-analysis aims to collect and provide robust evidence from observational comparative studies, to advance the understanding of disease progressing and the use of OCTA for management in patients with non-ocular BD.

## Methods

Current meta-analysis was conducted in accordance with the Preferred Reporting Items for Systematic reviews and Meta-Analyses (PRISMA) statement (12). Ethical approval and informed consent were not demanded. This study has been registered in Prospero database.

### Search strategy

Two independent reviewers (FSP and SXY) comprehensively searched the Pubmed, Embase, and Cochrane library for articles published form inception to February 2022, using the following terms: ((“Behcet’s” [tiab]) OR (“Behcet’s disease” [tiab]) OR (“Behcet syndrome” [tiab]) OR (“Behcet” [tiab]) OR (“Behcet disease” [tiab])) AND ((“OCTA” [tiab]) OR (“optical coherence tomography angiography” [tiab]) OR (“OCT-angiography” [tiab]) OR (“angio-OCT” [tiab])). The references of identified publications were reviewed thoroughly for potentially eligible investigations.

### Eligibility criteria

The included studies met the following criteria: (1) original comparative studies including non-ocular BD patients and control volunteers; (2) sample size of BD patients in each investigation was at least 10; (3) studies focusing on the macular microvasculature alterations rather than optic disc; (4) sufficient and clearly reported OCTA data to acquire weighted mean difference (WMD) for comparison. The exclusion criteria were as follows: (1) patients with other ocular disorders such as diabetic retinopathy, retinal vein occlusion and glaucoma etc.; (2) conference abstracts, case reports, animal experiments, review, editorials and comments; (3) studies did not provide the inclusion criteria to ensure the high quality and reliability of OCTA images.

### Data extraction and quality assessment

After removing duplicates, two reviewers (FSP and SXY) independently screened title and abstracts to exclude irrelevant articles and then reviewed full texts to evaluate eligible investigations. The following clinical demographics and characteristics were recorded: publication year, first author, sample size of BD patients and healthy individuals, geographic location, survey period, disease duration of BD, OCTA device, macular scan size, outcome variables of OCTA scan. Two independent researchers (FSP and SXY) extracted and retrieved data from the eligible studies. The criteria proposed by the Agency for Healthcare Research and Quality (AHRQ) was employed to assess the methodological quality of cross-sectional studies, which contained 11 items, with a better score indicating lower risk of bias. Any discrepancy was resolved through consensus with a third reviewer (LJ), if necessary.

### Quantitative analysis

Stata 12.0 (Stata Corp, College Station, TX, United States) was employed to perform statistical analyses. WMD with 95% confidence interval (CI) was used to estimate continuous outcomes in present meta-analysis. Heterogeneity among studies was evaluated with the *I*^2^-test. Fixed-effect model was applied when there was no significant heterogeneity among studies, otherwise, random-effect model was selected. Publication bias was assessed by Egger test. A *p* < 0.05 was considered statistically significant.

## Results

### Study inclusion

**FIGURE 1:**
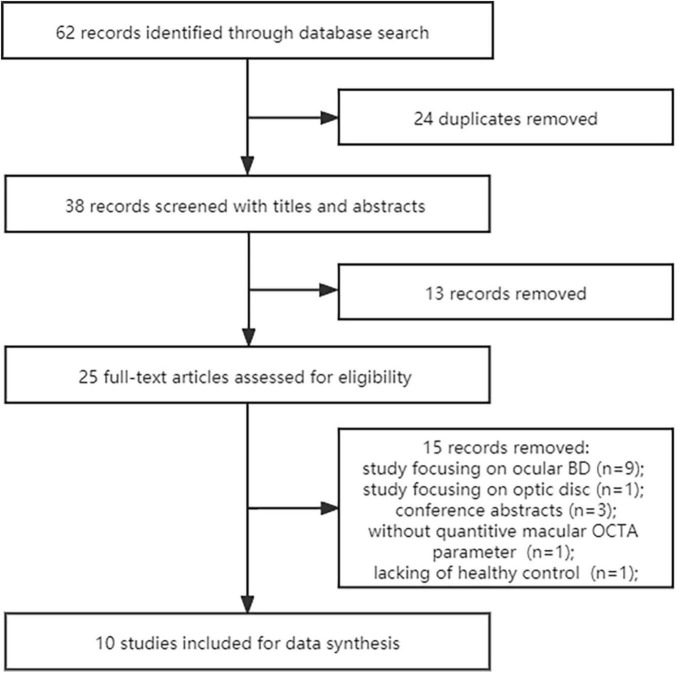
Flow diagram of selection process. *Is the length and width of macular scan size is 6 mm.

A total of 62 potentially eligible records were retrieved from Pubmed (*n* = 29), Embase database (*n* = 30) and Cochrane database (*n* = 3) from inception, of which 24 duplicates were removed. After screening titles and abstracts of each article, 25 studies remained for further evaluating. In addition, 9 investigations focusing on BD patients with clinical ocular manifestations, 1 record focusing on optic disc of BD, 3 conference abstracts, 1 article without sufficient data and 1 study without healthy control group were excluded. Therefore, as shown in Figure 1, **10** original cross-sectional investigations were eventually included for quantitatively synthesis in the present meta-analysis (8–10, 13–19).

Of these studies, 386 eyes in non-ocular BD and 418 eyes in healthy volunteers were assessed to determine the microvascular alterations. Taking the high prevalence of BD into consideration, most studies are performed in various ophthalmic centers of Turkey. Clinical characteristics and demographics were summarized in Table 1, including disease duration of BD, OCTA macular scan size and primary outcomes etc. OCTA parameters in nine studies were assessed using RTVue XR Avanti (Optovue, Inc., Fremont, CA), while one investigation using Spectralis OCT (Heidelberg Engineering, Heidelberg, Germany).

**TABLE 1:** Clinical demographics of included studies.

References	No. of eyes (BD/HC)	Country	Survey period	Disease duration (year)	OCTA device	Macular scan size	OCTA variables
Çömez et al. (8)	42/40	Turkey	NA	6.0 ± 5.1	Optovue	6*6 mm	FAZ, MVD
Goker et al. (10)	22/28	Turkey	2016–2018	10.4 ± 6.6	Optovue	6*6 mm	FAZ, MVD
Raafat et al. (13)	20/20	Egypt	2016–2016	12.7 ± 8.8	Optovue	3*3 mm	FAZ, MVD
Deǧirmenci et al. (14)	44/49	Turkey	NA	NA	Optovue	6*6 mm	FAZ, MVD
Karalezli et al. (9)	56/50	Turkey	2018–2019	7.4 ± 3.6	Optovue	6*6 mm	FAZ, MVD
Koca et al. (15)	51/53	Turkey	2016–2017	NA	Optovue	3*3 mm	MVD
Smid et al. (16)	23/22	Netherlands	2018–2019	11.0	Heidelberg	3*3 mm	FAZ
Yilmaz et al. (17)	40/60	Turkey	2018–2018	9.7 ± 7.3	Optovue	6*6 mm	FAZ, MVD
Küçük et al. (18)	56/61	Turkey	2020–2020	7.0 ± 4.0	Optovue	6*6 mm	FAZ, MVD
Simsek et al. (19)	32/35	Turkey	NA	7.1 ± 2.5	Optovue	6*6 mm	MVD

BD, Behcet’s disease; HC, Healthy control; FAZ, Foveal avascular zone; MVD, Macular vessel density; NA, Not applicable. *Is the length and width of macular scan size is 6 mm.

### Macular vessel densities in non-ocular Behcet’s disease compared with healthy controls

**FIGURE 2:**
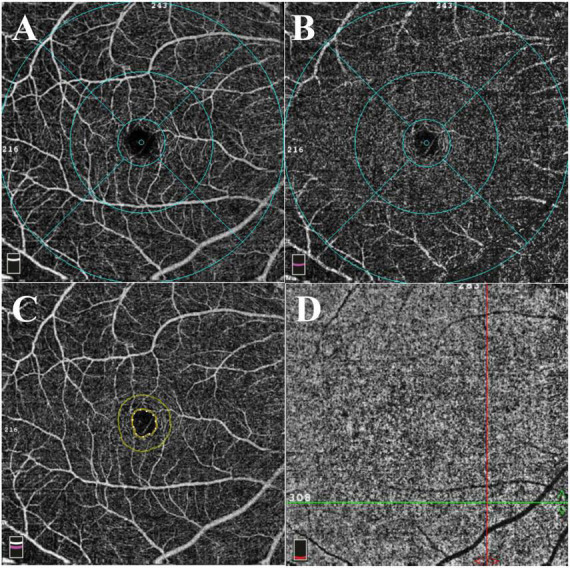
Representative 6*6 mm macular scan image of a healthy individual. **(A)** Superficial capillary plexus. The foveal region was defined as a 1 mm diameter circle centered on the fovea. The area between 1 and 3 mm diameter circle centered on the fovea was defined as parafoveal region. The perifoveal region was defined as the area between 3 and 6 mm diameter circle centered on the fovea. **(B)** Deep capillary plexus. **(C)** The foveal avascular zone (FAZ). The area and perimeter of FAZ were automatically calculated using installed software. Acircularity index was defined as the ratio between the measured perimeter and the perimeter of the same size circular area. **(D)** The flow area of choriocapillaris was calculated in a 3 mm radius circle centered on the fovea.

**FIGURE 3:**
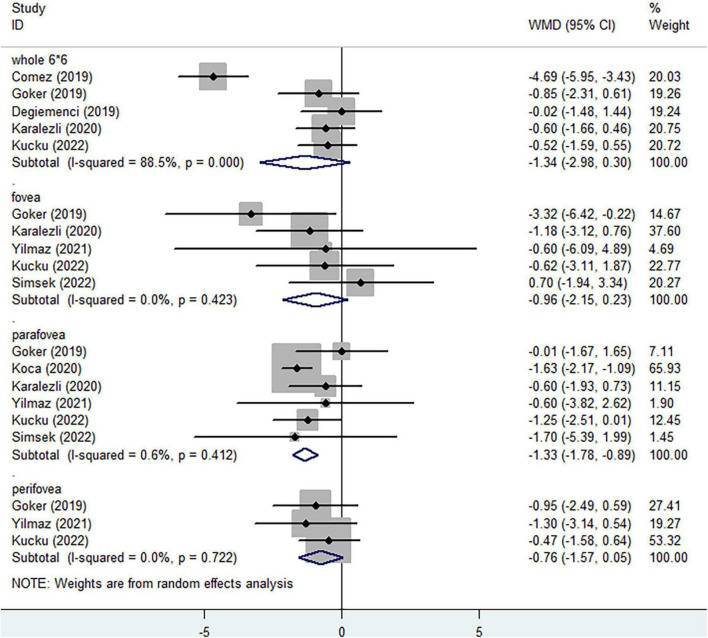
Forest plot of macular vessel density in the superficial capillary plexus (SCP) of non-ocular BD patients compared with healthy controls.

The vessel area divided by the region area was defined as vessel density of each region and the regions of interest are shown in Figure 2. In the SCP, regarding the whole enface of 6*6 mm scan image, non-ocular BD did not significantly decreased vessel density (Figure 3, WMD = −1.34, 95%CI: −2.98, 0.30; *I*^2^ = 88.5%), compared to healthy group. When considering foveal region, no significant reduced vessel density was determined in non-ocular BD (Figure 3, WMD = −0.96, 95%CI: −2.15, 0.23; *I*^2^ = 0%). In addition, significantly lower parafoveal vessel density was observed in non-ocular BD (Figure 3, WMD = −1.33, 95%CI: −1.78, −0.89; *I*^2^ = 0.6%). There was no significant difference in perifoveal area between non-ocular BD and healthy controls (Figure 3, WMD = −0.76, 95%CI: −1.57, 0.05; *I*^2^ = 0%).

**FIGURE 4:**
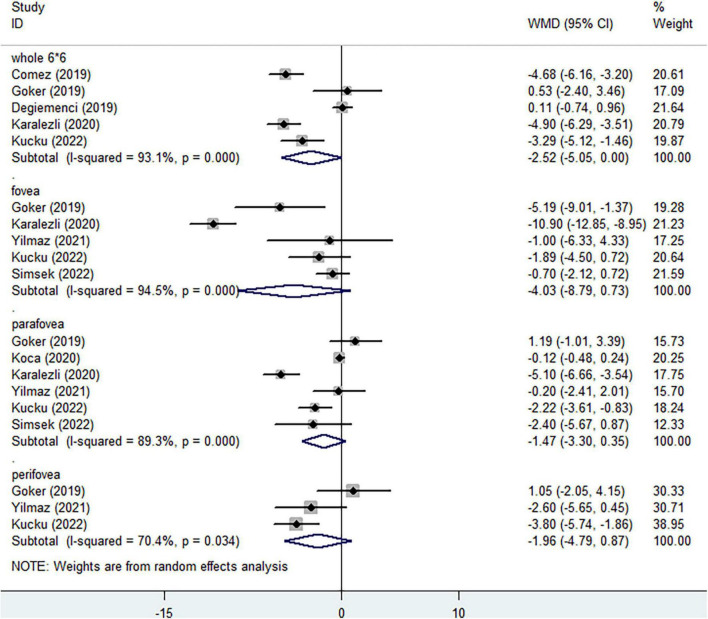
Forest plot of macular vessel density in the deep capillary plexus (DCP) of non-ocular BD patients compared with healthy controls.

In the DCP, when considering the whole enface of 6*6 mm scan image, lower vessel density was assessed in non-ocular BD, without statistical significance (Figure 4, WMD = −2.52, 95%CI: −5.05, 0.00; *I*^2^ = 93.1%). Compared with healthy individuals, no significantly decreased vessel densities were determined in foveal (Figure 4, WMD = −4.03, 95%CI: −8.79, 0.73; *I*^2^ = 94.5%) and parafoveal regions (Figure 4, WMD = −1.47, 95%CI: −3.30, 0.35; *I*^2^ = 89.3%). Three studies identified the reduced perifoveal vessel density in non-ocular BD, with no significant difference (Figure 4, WMD = −1.96, 95%CI: −4.79, 0.87; *I*^2^ = 70.4%).

### Comparisons of foveal avascular zone parameters and choriocapillaris between non-ocular Behcet’s disease and controls

**FIGURE 5:**
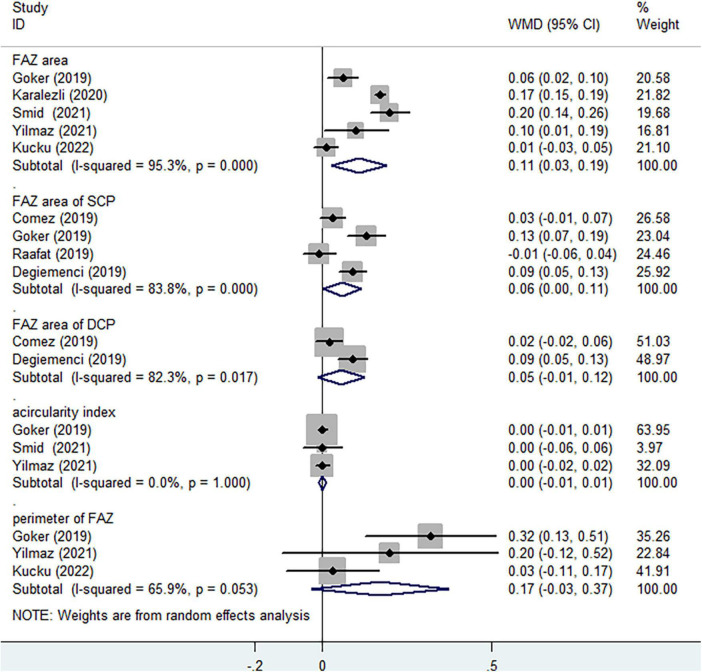
Forest plot of foveal avascular zone (FAZ) characteristics in non-ocular BD.

**FIGURE 6:**
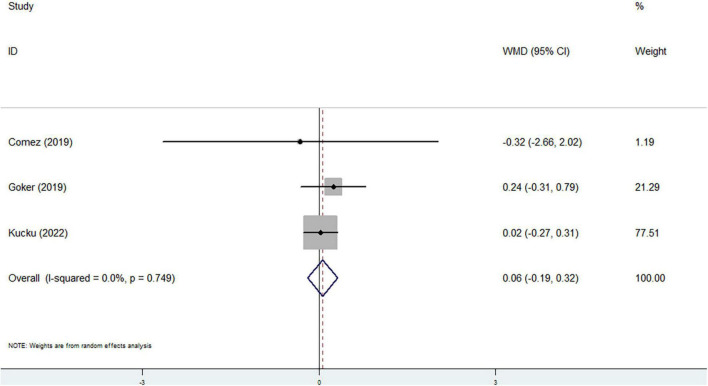
Forest plot of flow area of choriocapillaris layer at 3 mm radius area in non-ocular BD.

Several studies reported different clinical characteristics of FAZ, respectively. Significantly increasing FAZ area in non-ocular BD was observed in comparison to healthy controls (Figure 5, WMD = 0.11, 95%CI: 0.03, 0.19; *I*^2^ = 95.3%). Slightly expanded area of FAZ in SCP (Figure 5, WMD = 0.06, 95%CI: 0.00, 0.11; *I*^2^ = 83.8%) and DCP (Figure 5, WMD = 0.05, 95%CI: −0.01, 0.12; *I*^2^ = 82.3%) were identified in non-ocular BD, without significant difference. Increased acircularity index and perimeter of FAZ indicated the potential macular ischemia. No significant differences of the acircularity index (Figure 5, WMD = 0.00, 95%CI: −0.01, 0.01; *I*^2^ = 0%) and perimeter (Figure 5, WMD = 0.17, 95%CI: −0.03, 0.37; *I*^2^ = 65.9%) of FAZ were determined between non-ocular BD and healthy group. Considering the flow area of choriocapillaris layer at 3 mm radius area, no significant difference was observed (Figure 6, WMD = 0.06, 95%CI: −0.19, 0.32; *I*^2^ = 0%).

### Risk of bias and publication bias

As shown in Supplementary Table 1, the risk of bias evaluated by Agency for Healthcare Research and Quality of all included studies were relatively low, indicating high methodological quality and reliable results. No significant publication bias was determined in the present meta-analysis (Supplementary Table 2).

## Discussion

BD is a chronic autoinflammatory vasculitis with various systems and organs involved (20). As previous epidemiologic studies reported, the prevalence of BD varies 20–420 cases per 100,000 individuals in Turkey (21, 22). Despite the importance of early diagnosis of BD, there remains a paucity of effective means to establish definitive diagnosis after the onset of clinical manifestations. Thus, exploring sensitive and convenient markers for early diagnosis has been attracted increasing attention. OCTA is a novel, non-invasive and repeatable imaging tool, which has the ability to provide quantitative and elaborate determination of separate retinal and choroidal microvascular alterations (5, 23). Given that BD is one of the vascular occlusive syndromes, most studies found that ocular ischemia can still occur in patients with no obvious ocular manifestations. However, the results vary widely among different studies. Slightly decreased vessel densities of fovea and parafovea in non-ocular BD were observed in comparison to healthy individuals, while others demonstrated significantly reduced macular perfusion in corresponding regions (9, 10, 18, 19). To elucidate the confusing data, this meta-analysis was performed to identify the retinal and choroidal characteristics of non-ocular BD.

The most obvious result emerging from this meta-analysis is that the vessel densities of the SCP and DCP reduced in non-ocular BD in comparison with healthy controls, especially in the parafoveal region of SCP. Although the detailed mechanism remains unknown, the reduction of vessel density in BD probably could be attributed to diffuse occlusive vasculitis, which is caused by the circulation of endothelial cell proliferation and pathological immunocomplexes (24). These results are essential in respect of indicating that retinal microvascular alterations appear before the emergence of ocular manifestations. Previous studies found higher choroid thickness in non-ocular BD, indicating the potential of subclinical choroidal inflammation (8, 19). Moreover, significantly increased serum levels of resistin, tumor necrosis factor-α and lnterleukin-6 have been demonstrated in non-ocular BD, indicating the deviation of inflammatory and anti-inflammatory balance (25, 26). Smid and colleagues hypothesized that the preclinical retinal microvasculature alteration may predict further development of non-ocular BD and first uveitis attack (16). Longitudinal cohort studies in the future are needed to validate the hypothesis. In a word, detection of retinal capillary plexus with OCTA contribute to evaluate the retinal structures and disease status of BD patients without ocular signs and symptoms.

A large number of studies have demonstrated the FAZ is an effective predictor in retinal disorders and uveitis (27–29). In this analysis, we found increased FAZ area in full plexus and separate segmentations, indicating macular ischemia in non-ocular BD. We speculate that FAZ area in full retinal vasculature, could be considered as a sensitive from of ocular involvement for non-ocular BD patients. In this meta-analysis, no decreased flow area of choriocapillaris was determined. A possible explanation is only three studies have reported the data of choriocapillaris, limiting our ability to draw definitive conclusion. It is also worth mentioning that the penetrating power of spectral domain OCTA limits the detecting and measuring choroid exactly. The application of swept-source OCTA may generate fresh insights into choroidal vascular abnormalities in BD (30).

There remains substantial heterogeneity in multiple analyses of retinal macular perfusion, which could be attributed to several possible explanations. First, the demographics of recruited BD patients varies widely among studies in the present meta-analysis, resulting in significant clinical heterogeneity. In addition, the OCTA images and parameters are obtained and calculated from different OCTA devices and algorithm, which may cause heterogeneity. Different scan quality of each image and various techniques of segmentation, also potentially accounting for the high heterogeneity. Moreover, it is worthwhile to point out that both eyes of participants are included in several studies, which may lead to potential false positive results and statistical heterogeneity in this analysis.

There are several limitations in this analysis should be considered. First, all of the included studies were cross-sectional, which might result in selection bias. Thus, these results require validation in multi-center studies. Second, essential clinical characteristics of patients such as disease activity and currently treatment status were not reported, limiting further comparisons and investigations. Despite these limitations, the potency of this meta-analysis is that the FAZ features including area in various segmentations, acircularity index and perimeter of FAZ are quantitatively measured comprehensively in non-ocular BD for first time.

In conclusion, this meta-analysis demonstrated reduced parafoveal vessel density of SCP and larger FAZ area in full retinal vasculature might be potentially effective markers in evaluating microvascular alterations in BD patients without ocular manifestations. Further prospective studies should be conducted to clarify the role of OCTA in assessing disease activity, detecting potential complications and evaluating clinical outcomes in non-ocular BD.

## Data availability statement

The original contributions presented in this study are included in the article/Supplementary Material, further inquiries can be directed to the corresponding author/s.

## Author contributions

SF and XS contributed to the design of review and extracted the data. ZC and XL contributed to analysis of data. SF drafted the manuscript. SY and JL critically reviewed it and suggested amendments prior to submission. All authors contributed to the article and approved the submitted version.

## Conflict of interest

The authors declare that the research was conducted in the absence of any commercial or financial relationships that could be construed as a potential conflict of interest.

## Publisher’s note

All claims expressed in this article are solely those of the authors and do not necessarily represent those of their affiliated organizations, or those of the publisher, the editors and the reviewers. Any product that may be evaluated in this article, or claim that may be made by its manufacturer, is not guaranteed or endorsed by the publisher.
