# Supplementary material for: Retinal and choroidal microvascular alterations in Behcet’s disease without ocular manifestations: A systematic review and meta-analysis
Source: Front Med (Lausanne). 2022 Jul 22;9:911990. doi: 10.3389/fmed.2022.911990 (PMC9353174; doi:10.3389/fmed.2022.911990)
Supplement: Supplementary file 1 [file Table_1.DOCX]

Supplementary Material

**Table S1** Methodological quality of included studies

| Study | Items of assessments | | | | | | | | | | |
| --- | --- | --- | --- | --- | --- | --- | --- | --- | --- | --- | --- |
|  | 1 | 2 | 3 | 4 | 5 | 6 | 7 | 8 | 9 | 10 | 11 |
| Comez, 2019 | Y | Y | N | ? | NA | Y | ? | Y | NA | Y | NA |
| Goker, 2019 | Y | Y | Y | Y | NA | Y | Y | Y | NA | Y | NA |
| Raafat, 2019 | Y | Y | Y | Y | NA | Y | Y | Y | NA | Y | NA |
| Degiemenci, 2020 | Y | Y | N | ? | NA | Y | Y | Y | NA | Y | NA |
| Karalezli, 2020  Koca, 2020  Smid, 2020 | Y  Y  Y | Y  Y  Y | Y  Y  Y | Y  Y  Y | NA  NA  NA | Y  Y  Y | Y  Y  Y | Y  Y  Y | NA  NA  NA | Y  Y  Y | NA  NA  NA |
| Yilmaz, 2021  Kucuk, 2022  Simsek, 2022 | Y  Y  Y | Y  Y  Y | Y  Y  N | Y  Y  ? | NA  NA  NA | Y  Y  Y | Y  Y  Y | Y  Y  Y | NA  NA  NA | Y  Y  Y | NA  NA  NA |

Y=yes; N=no; ?=unclear; NA=Not applicable

Studies fulling the criteria recommended by the Agency for Healthcare Research and Quality: (1) Define the source of information (survey, record review); (2) List inclusion and exclusion criteria for exposed and unexposed subjects (cases and controls) or refer to previous publications; (3) Indicate time period used for identifying patients; (4) Indicate whether or not subjects were consecutive if not population-based; (5)  Indicate if evaluators of subjective components of study were masked to other aspects of the status of the participants; (6) Describe any assessments undertaken for quality assurance purposes (e.g., test/retest of primary outcome measurements); (7) Explain any patient exclusions from analysis; (8) Describe how confounding was assessed and/or controlled; (9) If applicable, explain how missing data were handled in the analysis; (10) Summarize patient response rates and completeness of data collection; (11) Clarify what follow-up, if any, was expected and the percentage of patients for which incomplete data or follow-up was obtained.

**Table S2** Publication bias of OCTA parameters (number of included studies≥4)

| Outcome variables | Number of studies | Egger test (p value) |
| --- | --- | --- |
| Macular VD (%)  SCP  Whole 6*6 mm  Fovea  Parafovea  DCP  Whole 6*6 mm  Fovea  Parafovea  FAZ  FAZ area  FAZ area of SCP | 5  5  6  5  5  6  5  4 | 0.406  0.909  0.169  0.464  0.853  0.301  0.406  0.677 |

SCP: Superficial capillary plexus; DCP: Deep capillary plexus;

FAZ: Foveal avascular zone
